# Novel Selectable Marker Sesquiterpenoid Antibiotic Pentalenolactone

**DOI:** 10.3390/ijms252413328

**Published:** 2024-12-12

**Authors:** Arina A. Nikandrova, Anna D. Petriakova, Anton R. Izzi, Garegin A. Petrosyan, Vadim N. Tashlitsky, Vera A. Alferova, Tatiana V. Panova, Maria G. Khrenova, Mikhail V. Biryukov, Yuliya V. Zakalyukina, Maria I. Zvereva, Dmitrii A. Lukianov, Petr V. Sergiev

**Affiliations:** 1 Center for Molecular and Cellular Biology, 121205 Moscow, Russia; arinanikandrova@mail.ru (A.A.N.); antonizzi5@mail.ru (A.R.I.); petya@genebee.msu.ru (P.V.S.); 2 Department of Biology, Lomonosov Moscow State University, 119991 Moscow, Russia; g.greysilver@yandex.ru (A.D.P.); metrim@gmail.com (M.V.B.); 3 Belozersky Institute of Physico-Chemical Biology, Lomonosov Moscow State University, 119991 Moscow, Russia; alferovava@gmail.com; 4 School of Bioengineering and Bioinformatics, Lomonosov Moscow State University, 119991 Moscow, Russia; 5 Department of Soil Science, Lomonosov Moscow State University, 119991 Moscow, Russia; gareg2004@list.ru (G.A.P.); juline@mail.ru (Y.V.Z.); 6 Department of Chemistry, Lomonosov Moscow State University, 119991 Moscow, Russia; tashlitsky@belozersky.msu.ru (V.N.T.); tvk@genebee.msu.ru (T.V.P.); wasabiko13@gmail.com (M.G.K.); maria.i.zvereva@yandex.ru (M.I.Z.); 7 Shemyakin-Ovchinnikov Institute of Bioorganic Chemistry, Russian Academy of Science, 117997 Moscow, Russia; 8 Bach Institute of Biochemistry, Federal Research Centre “Fundamentals of Biotechnology” of the Russian Academy of Sciences, 119071 Moscow, Russia; 9 Center for Translational Medicine, Sirius University of Science and Technology, 354340 Sochi, Russia

**Keywords:** antibiotics, selectable marker, pentalenolactone

## Abstract

Antibiotic resistance has been and remains a major problem in our society. The main solution to this problem is to search and study the mechanisms of antibiotic action. Many groups of secondary metabolites, including antimicrobial ones, are produced by the *Actinomycetota* phylum. The actinobacterial strains isolated from habitats that have not been well studied are of great interest. Due to high resource competition, antibiotics are now considered a ‘trump card in the game of life’ due to their presence in natural substrates with limited nutrients. Potentially, strains isolated from such habitats can be producers of novel or poorly studied antibiotics. In the current research, we identified the strain *Streptomyces* sp. AP22 from the soils of the Akhshatyrsky Gorge, which is capable of producing pentalenolactone. This study describes the phenotypic and morphological characteristics of *Streptomyces* sp. AP22 and its biological activity. Pentalenolactone is a known inhibitor of glyceraldehyde-3-phosphate dehydrogenase (GAPDH), an important enzyme involved in glycolysis. We identified a previously unknown mutation in the *gapA* gene encoding glyceraldehyde-3-phosphate dehydrogenase that confers resistance to this antibiotic compound. This antibiotic is not used in clinical practice, so its application as a selectable marker will not lead to the creation of pathogens resistant to clinically relevant antibiotics. In this case, the selectable marker is based on a genetic construct containing the glyceraldehyde-3-phosphate dehydrogenase gene with a resistance mutation. The use of this selectable marker can be applied to various genetic and molecular techniques, such as cloning and transformation. This can help to facilitate genetic and molecular biology studies of strains resistant to standard selectable markers such as kanamycin or ampicillin.

## 1. Introduction

Antibiotic resistance in pathogenic bacteria is now a critical international issue, which makes it urgent to isolate and study new microorganisms producing secondary metabolites. The antibiotic resistance crisis is caused by various factors, including the overuse and misuse of antibiotics as well as their widespread uncontrolled use in agriculture and animal husbandry [1].

Actinomycetes are a group of Gram-positive bacteria of the phylum *Actinomycetota*, and the genome contains a high number of GC pairs. This group is widely known as a provider of active secondary metabolites, synthesizing about two-thirds of all naturally occurring antibiotics currently in use, as well as many antitumor, anti-inflammatory, and antifungal compounds [2].

Actinomycetes are producers of the following classes of antibiotics used in clinical practice: aminoglycosides, tetracyclines, macrolides, glycopeptides, cycloserines, carbapenems, amphenicols, lincosamides, etc. [3]. Thus, antibiotics synthesized by actinomycetes are characterized by a significant diversity of chemical structures, which accounts for a wide range of their biological activity. Moreover, the study of actinomycete metabolism can contribute to solving not only medical problems but also the challenges faced by agriculture, pharmaceuticals, and other fields [4].

Extensively drug-resistant strains, usually isolated from infected patients, are already resistant to traditional selectable genetic markers, which limits the possibility of pathogenesis studies through genetic disruption. Therefore, the introduction and optimization of selectable markers is crucial to introduce fundamental molecular biology techniques for use against these strains.

The sesquiterpenoids include a family of tricyclic compounds, the pentalenolactones. This group, unique in chemical structure, is a metabolite of numerous members of the genus *Streptomyces*. Pentalenolactone was first isolated in the mid-1950s in Great Britain from the microorganism *Streptomyces roseogriseus*, and for some time was known as Arenemycine, Arenaemycin E, or PA-132 [5,6]. The inhibition of glyceraldehyde-3-phosphate dehydrogenase (GAPDH) is the mechanism of action of pentalenolactone. Mutations of this gene gain resistance to this antibiotic.

## 2. Results

### 2.1. Isolation of Actinobacteria Strains and Screening Antimicrobial Activity

Due to the need to search for antibiotic-producing strains, poorly studied ecosystems and habitats are relevant sources of substrates for strain isolation.

About 10 strains were isolated from a soil sample collected in the Akhshtyrsky Gorge, but only 6 of them showed inhibitory activity against *E. coli*. The most interesting strain was AP22. In view of the increasing problem of the detection of many already known antibiotics in screening actinomycetes from classical habitats, it seems necessary to search for microorganisms in poorly studied ecosystems [7,8].

The Akhshtyr Gorge is an example of such an ecosystem. This gorge is located within Sochi National Park. The vast territory, little explored by producers, is characterized by a unique microclimate. The close flow of the Mzymta River led to the appearance of rapids and waterfalls, which contributed to the formation of caves and cavities. The increased humidity has led to a wealth of vegetation, and the gorge is composed of calcareous rocks—which are favorable factors for the existence of actinomycetes.

**Figure 1:**
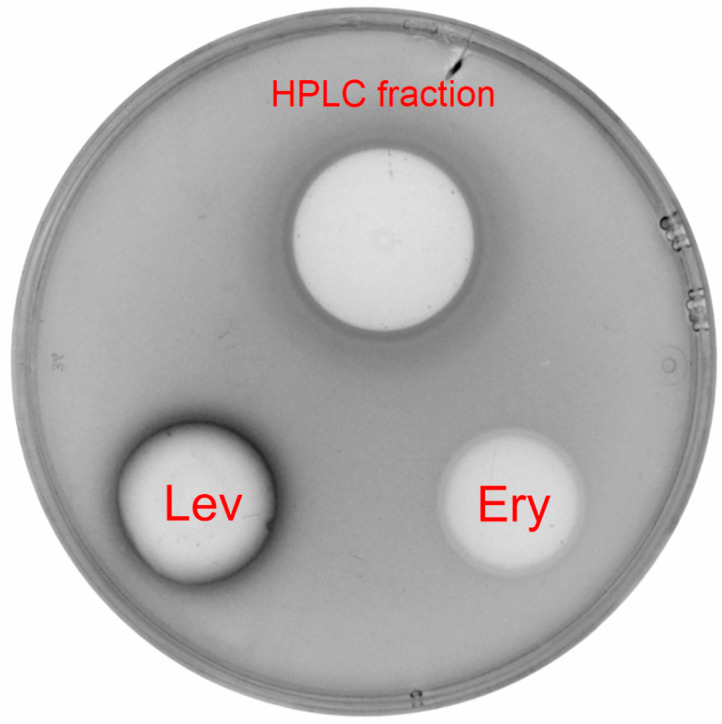
In vitro test for the antagonistic activity of the HPLC fraction on *E. coli* strain JW5503 ΔtolC. Erythromycin (Ery, 5 ug) and levofloxacin (Lev, 25 ng) were used as positive controls.

The antibacterial activity of the strains was evaluated using a hypersensitive *E. coli* ΔtolC strain transformed with a pDualrep2 reporter plasmid [9]. In the first stage, the *Streptomyces* sp. AP22 was cultivated on different solid media, and agar plugs were used to study the activity; afterward, the optimized liquid medium was prepared, and after 7 days of incubation, a cultural broth also showed activity. The activity of the HPLC purified sample was confirmed using a spot diffusion method (Figure 1). Inhibition zones on a petri dish covered with *E. coli* JW5503 ΔtolC cells represent the activity of pentalenolactone and control compounds.

### 2.2. Genome Features and Phylogenomic Analysis of Streptomyces sp. Strain AP22

Sequencing of the isolated DNA was carried out by Oxford Nanopore sequencing technology for genomic DNA by ligation by using the SQK-LSK109 protocol (Oxford Nanopore). The reads were assembled with Flye 2.9 [10]. The assembled genome was annotated with Prokka Version 1.14.6 [11]. Then, 16S rRNA and five housekeeping genes, DNA gyrase subunit B (gyrB), DNA-directed RNA polymerase subunit beta (rpoB), Tryptophan synthase beta chain (trpB), bacterial DNA recombination protein (recA), and ATP synthase subunit beta (atpD), were used for MLST [12].

**Figure 2:**
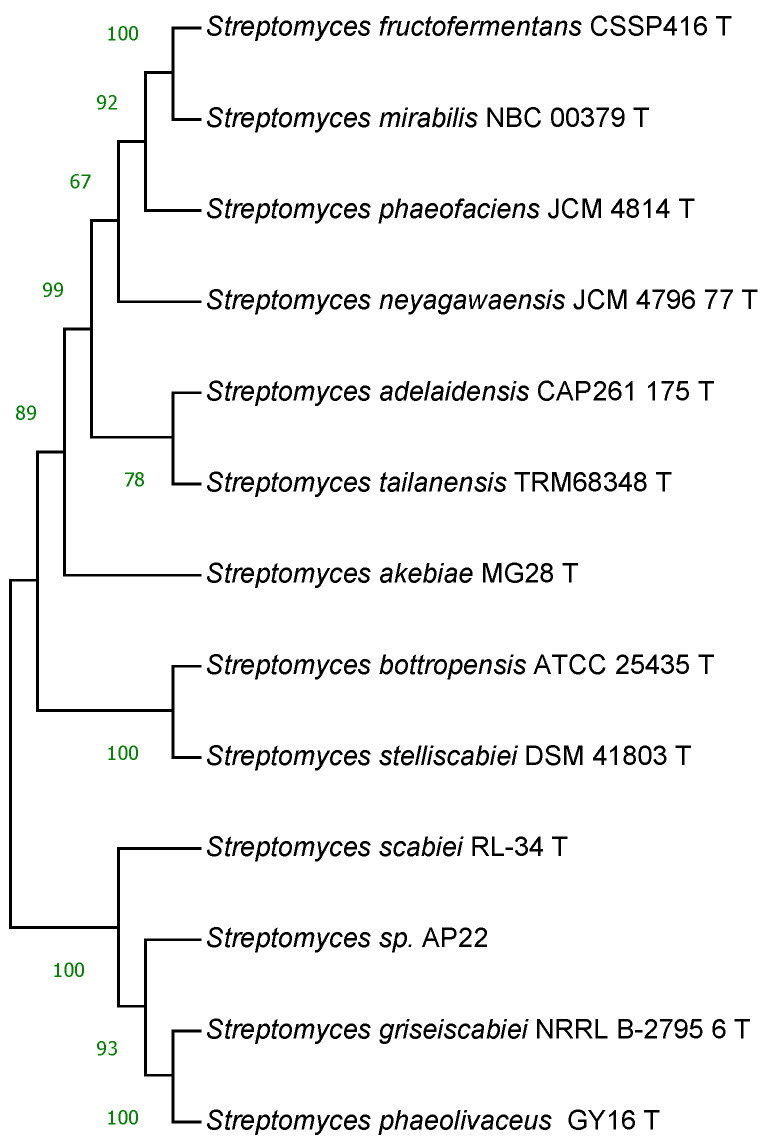
Phylogenetic tree based on six concatenated housekeeping gene sequences (16S rRNA, atpD, gyrB, recA, rpoB, trpB, *Streptomyces* sp. AP22, and related type strains) performed using neighbor-joining maximum-likelihood tree-making algorithms after CLUSTAL W alignment [11] by using MEGA software version XI [12].

Based on a phylogenetic analysis by 16S rRNA, *Streptomyces* sp. AP22 is close to *Streptomyces scabiei*, but MLST allows it to be grouped with two recently described species *Streptomyces griseiscabiei* and *Streptomyces phaeolivaceus* (Figure 2).

### 2.3. Phenotypic Characterization of Streptomyces sp. Strain AP22

**Figure 3:**
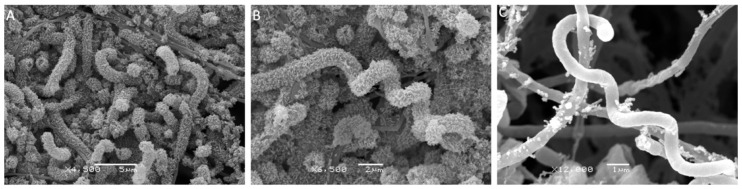
Scanning electron micrograph of the strain *Streptomyces* sp. AP22, showing the spore surface after incubation on ISP 3 medium at 28 °C for 14 days. Microphotographs show that most hyphae have a spiral shape. Some hyphae have a smooth surface, but the predominant majority of the aerial mycelium becomes warty and rough, due to the fact that it is covered with special surface proteins where spore-bearing hyphae are formed. Various magnification options are shown in the figure: (**A**) ×4500 (**B**) ×6500, and (**C**) ×12,000.

Electron microscopy was used for a more detailed morphological description (Figure 3), namely, a detailed determination of the structure of aerial mycelium. The sporophores of this strain are long, regular stretched spirals. The surface of the sporophore is rough, and no separation into spores was observed at the time of microscopy (14 days).

The two species (Table 1) were found to be the most closely related on the 16S basis. Their phenotypic characteristics on the same ISP media were similar, but the shape and surface of the sporophores were different. It should be noted that *Streptomyces* scabiei is a pathogen that affects agricultural crops, and the most well-studied metabolite of this species is Thaxtomin A.

### 2.4. Identification of the Active Compound and Its Confirmed Mechanism of Action

**Figure 4:**
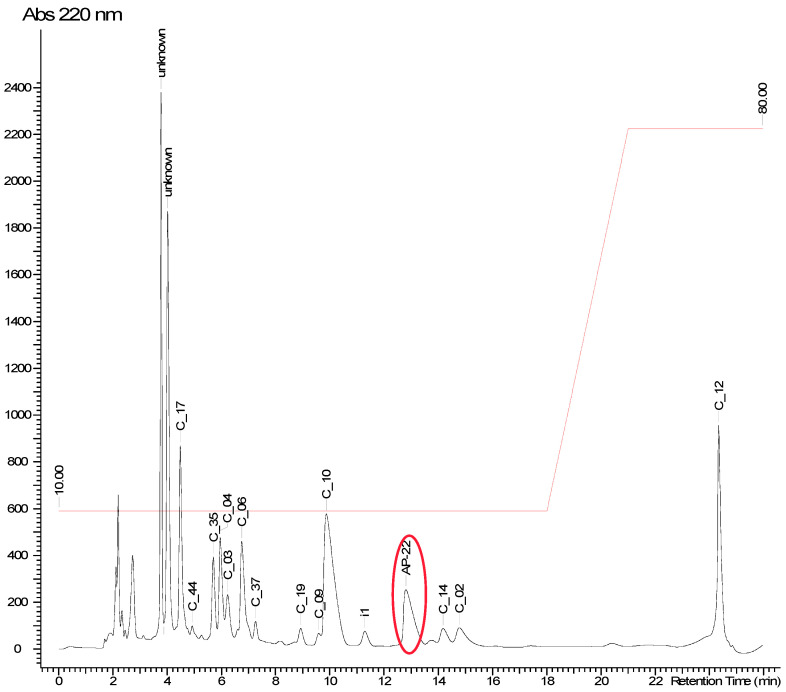
Chromatogram of the most active LPS fraction (10% acetonitrile). The active peak is marked by a red circle.

**Figure A1:**
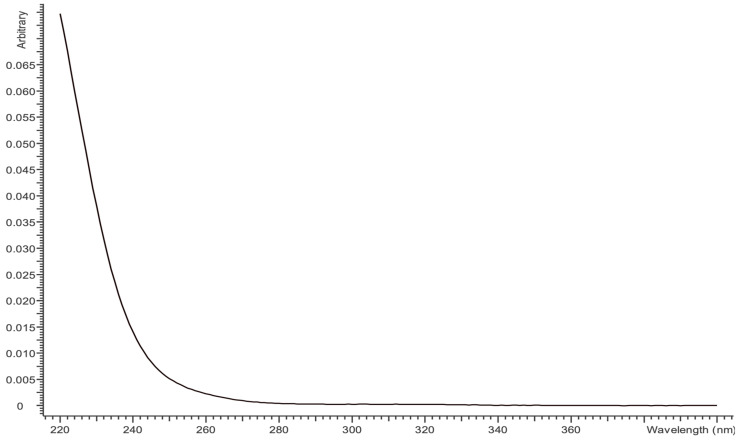
UV spectrum of the peak after HPLC purification of the most active LPS fraction (10% acetonitrile).

The obtained active fraction, isolated by solid-phase extraction, was subjected to additional fractionation and purification using high-performance liquid chromatography with a reversed-phase column. The method of purification was developed with ACD/AutoChrom software (2020.1.1). Optimal conditions were found: column Luna C18(2), 5 μm, 4.6 × 250 mm (Phenomenex, Torrance, CA, USA), isocratic elution 10% of MeCN, 10 mM ammonium acetate, for 18 min, followed by a gradient of up to 80% acetonitrile in 3 min, 25 °C, 1 mL/min (Agilent 1260, DAD, Santa Clara, CA, USA). Figure 4 shows the chromatogram, and the peak of the active compound is marked by a red circle; its UV spectra can be found in Figure A1.

**Figure 5:**
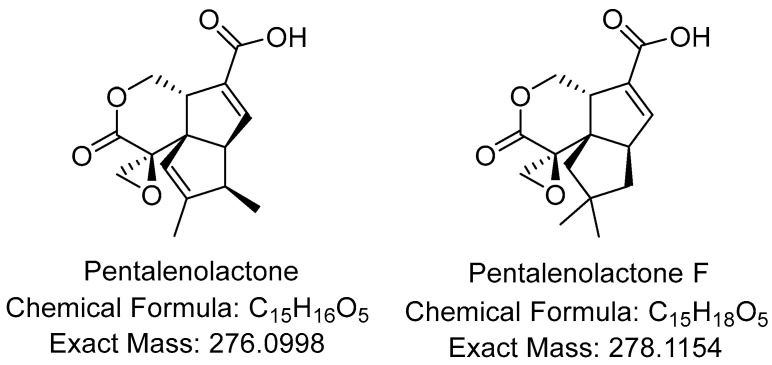
Proposed structures of the isolated compounds.

**Figure 6:**
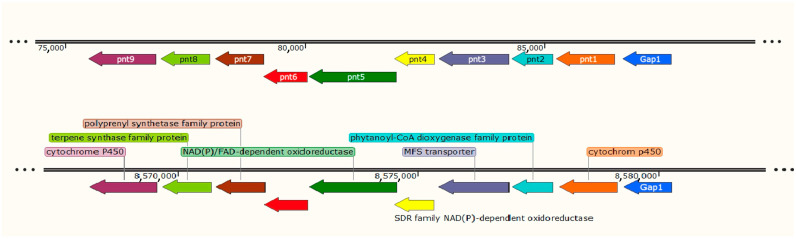
Genetic organization of pentalenolactone synthesis cluster in producer *Streptomyces* sp. AP22 (**top**) and *Streptomyces* sp. NBC_01231 (**bottom**). Homologous genes filled with the same colors, GenBank accession numbers are listed in Data Availability Statement.

**Figure A2:**
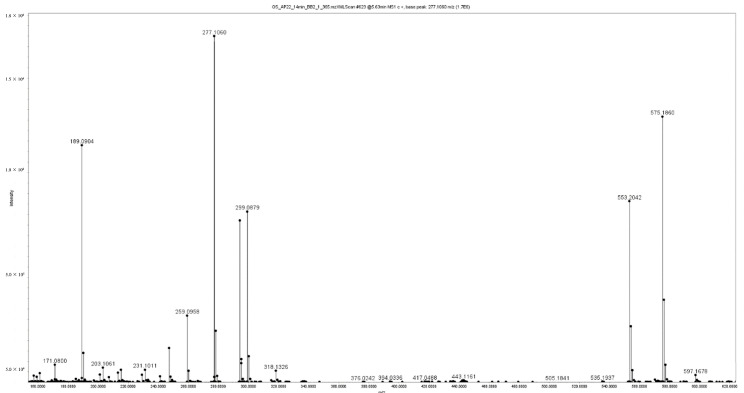
Mass spectrum (positive) of the compound with retention time 5.63 min.

**Figure A3:**
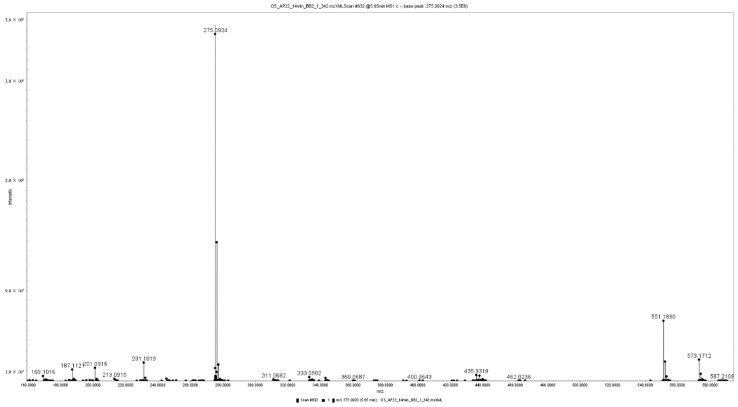
Mass spectrum (negative) of the compound with retention time 5.63 min.

**Figure A4:**
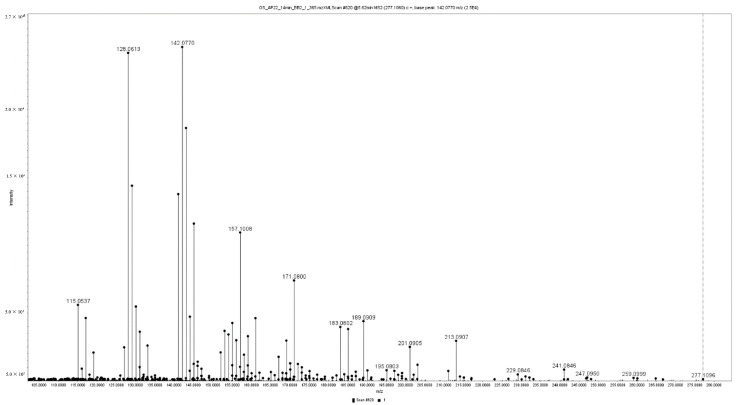
Fragmentation of [M + H]^+^ ion with *m*/*z* 277.106 (pentalenolactone).

**Figure A5:**
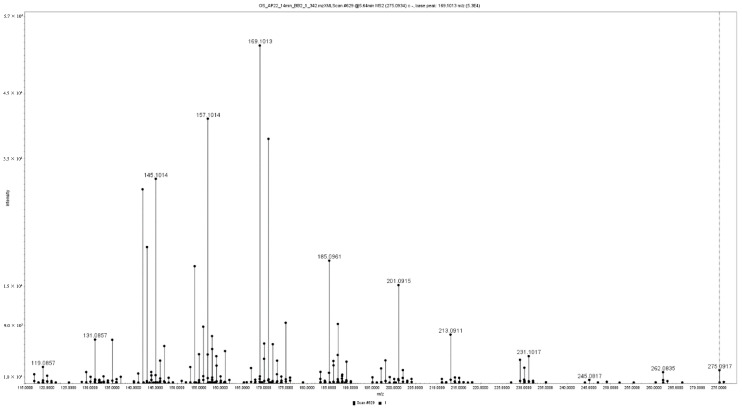
Fragmentation of [M − H]^−^ ion with m/z 275.092 (pentalenolactone).

**Figure A6:**
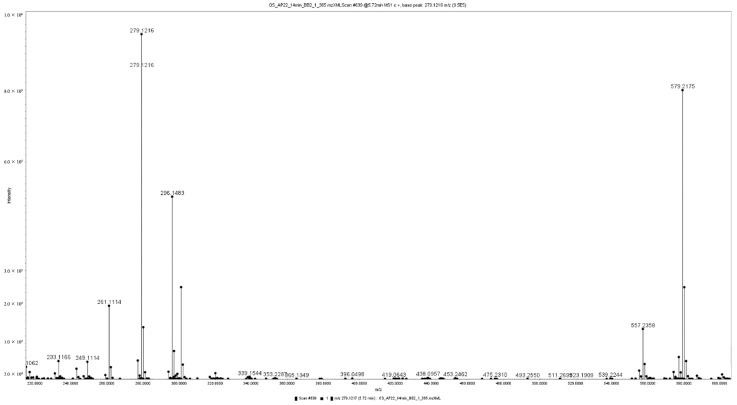
Mass spectrum (positive) of the compound with retention time 5.72 min.

**Figure A7:**
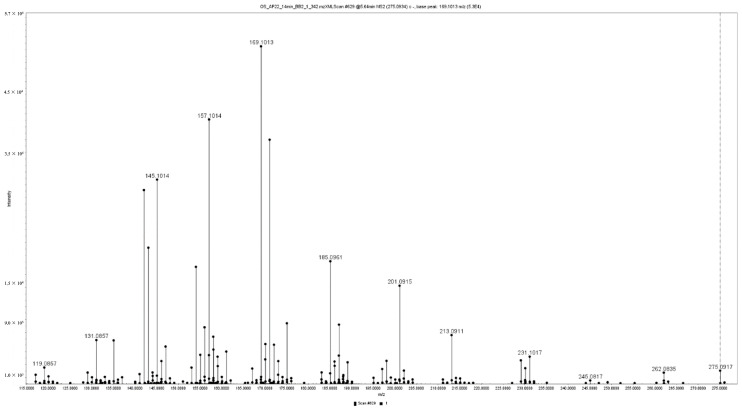
Mass spectrum (negative) of the compound with retention time 5.72 min.

**Figure A8:**
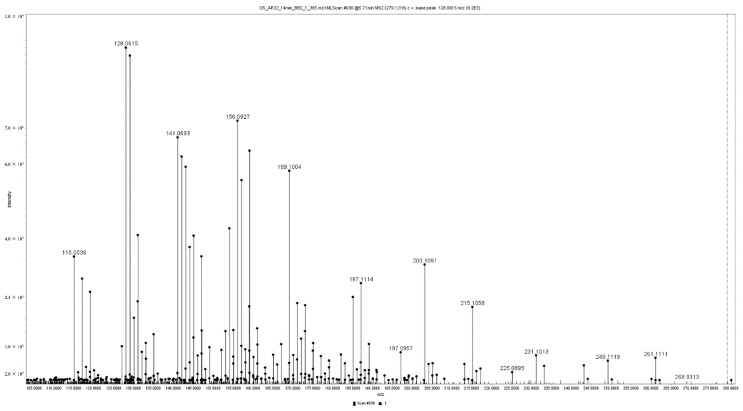
Fragmentation of [M + H]^+^ ion with *m*/*z* 279.122 (pentalenolactone F).

**Figure A9:**
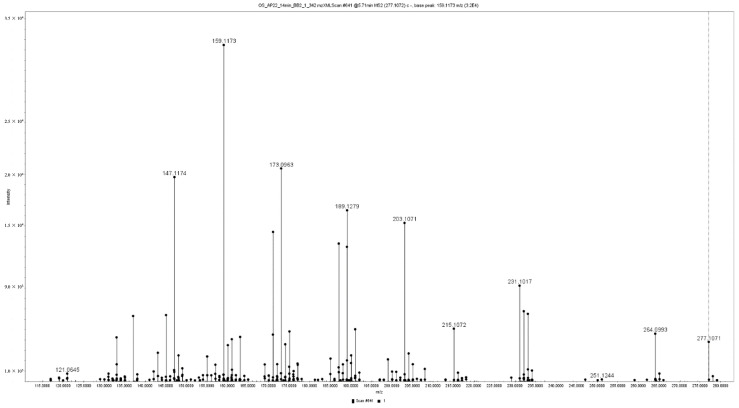
Fragmentation of [M − H]^−^ ion with *m*/*z* 277.107 (pentalenolactone F).

HR-LCMS analysis of the active fraction revealed two components with exact masses 276.0988 Da and 278.1142 Da, corresponding to molecular formulae C_15_H_16_O_5_ (Δ 3.5 ppm) and C_15_C_18_O_5_ (Δ 4.4 ppm). Fragmentation patterns are characteristic of sesquiterpene lactones (95% identity by CANOPUS [13] part of Sirius [14]). An in silico fragmentation analysis using MetFrag [15] revealed pentalenolactone and pentalenolactone F (Figure 5) as the most plausible hits. The UV-V spectrum (Figure 6) and fragmentation pattern (Figure A2, Figure A3, Figure A4, Figure A5, Figure A6, Figure A7, Figure A8 and Figure A9) correlate with the GC-MS data reported previously for the pentalenolactone methyl ester [16].

### 2.5. Analysis of Streptomyces sp. AP22 Bioactive Compound Biosynthetic Gene Clusters

A Blastn of the pentalenolactone cluster using nanopore sequencing showed the results of the producer strain (*Streptomyces* sp. AP22) as the database revealed the presence of the cluster in the fourth contig. The middle zone of this contig was blasted again on the core nucleotide database, resulting in *Streptomyces* sp. NBC_01231 (CP108521.1) as the closest hit. The strain genome processing using the antiSMASH program revealed a biosynthetic gene cluster of pentalenolactone, consisting of nine main genes and resistant to the pentalenolactone Gap gene. After the alignment of the components of this cluster (including the Gap gene) with the fourth contig, it was revealed that the genes in the producer strain have the same length and the same direction as in the case of the *Streptomyces* sp. NBC_01231 strain, which indicates the homology of the entire pentalenolactone biosynthesis cluster (Figure 6).

### 2.6. Selection of Resistant Clones

After resistant clones were selected, the minimum inhibitory concentration for these clones was determined. As a result, clones eight times more resistant to pentalenolactone were obtained (Table 2). Genomic DNA from these clones was isolated to determine the mutations leading to resistance.

**Figure A10:**
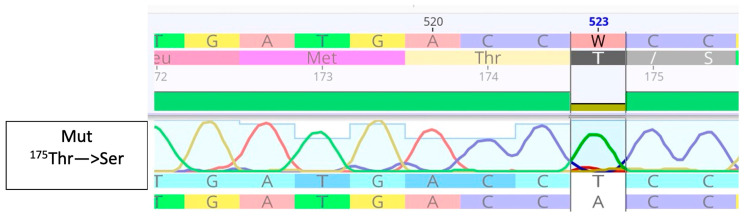
The *gapA* gene contains a replacement fragment, which resulted in a chromatogram from a Sanger capillary sequencer for a mutant strain.

A full-genome sequence of the pentalenolactone-resistant *E. coli* dtolC strain was obtained using Oxford Nanopore sequencing. The results revealed a transition A–T at position 523 in the *gapA* gene sequence, so the threonine residue at position 175 in the protein was replaced by serine. Additionally, this mutation was also confirmed by Sanger sequencing (Figure A10).

The structures of wild-type and mutated GapA proteins were constructed using the AlphaFold program and were aligned in order to find differences that can be responsible for the appearance of pentalenolactone resistance.

**Figure 7:**
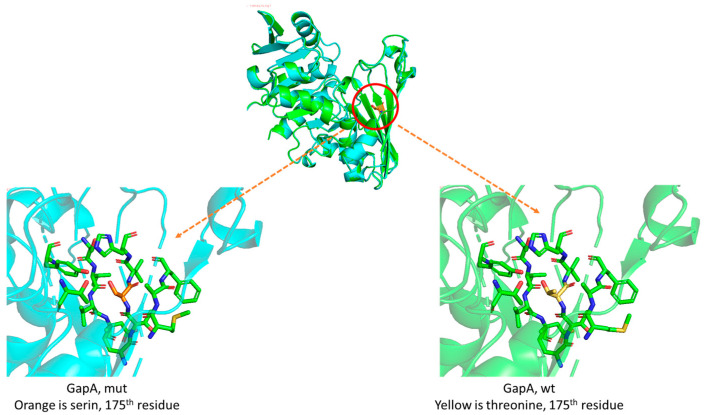
Alignment of wild-type and resistant-to-pentalenolactone mutated GapA protein structures. The location of the replacement is indicated by the red circle.

According to the results, the mutated GapA structure did not have any conformational changes that could explain why *E. coli* became tolerant to pentalenolactone when threonine was substituted with serine. The absence of a methyl group in the residue (Figure 7) was the only difference that could be detected.

**Figure 8:**
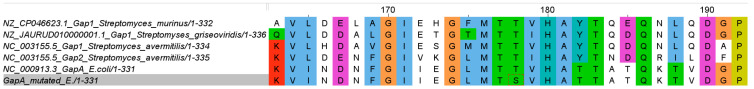
Alignment of 6 different protein sequences of bacterial glyceraldehyde-3-phosphate dehydrogenase. Red color indicates positively charged amino acid residues, purple—negatively charged, green—polar, orange—glycines, blue—hydrophobic, and cyan—aromatic. In mutated *E. coli* GapA protein, threonine at alignment position 178 (in *E. coli* protein sequence the position is 175) is replaced by serine, and this mutation makes bacteria resistant to pentalenolactone. What is more, the mutation cannot be observed in pentalenolactone producer species (such as *S. griseoviridis* and *S. gvermitilis*). The multiple sequence alignment was performed using the CLUSTALW program, and the results were visualized and edited using the Jalview program.

Alignment of different protein sequences of the Gap gene. The Gap1 isoform from pentalenolactone producers (*Streptomyces griseoviridis* and *Streptomyces avermitilis*) was resistant to the antibiotic, whereas the Gap2 isoform was not, as well as *E. coli* wild-type GapA and Streptomyces murinus Gap1. Resistant to pentalenolactone, the *E. coli* strain had only one mutation in the GapA gene at alignment position 178 (in *E. coli* protein sequence position 175), where Thr was replaced by Ser (Figure 8). Moreover, despite the fact that Streptomyces species were producers and had Gap1 isoforms resistant to pentalenolactone, this replacement was not discovered. This indicates that the *E. coli* GapA mutation at position 175 seems to be a previously undescribed phenomenon.

### 2.7. New Resistant Marker Verification

Based on pRFPCER, we prepared a construction containing two resistant markers. First, the original β-lactamase gene is under the ampR promoter [17]. Second, the *gapA*^mut^ gene contains the T175S substitution in the *gapA* gene, which resulted in resistance to pentalenolactone. This gene was inserted under the native T5 promoter [17].

We first determined the MIC of pentalenolactone against the *E. coli* DH5α cell line to verify its potential as a selective marker. The MIC of pentalenolactone against *E. coli* DH5α was 50 μg/mL. Our plasmid pCDF_PLR and control plasmid pRFPCER were used to transform *E. coli* DH5α cells. After that, we transferred these cells onto plates. First, plate with pentalenolactone with a concentration of 1000 μg/mL, which is 20 MIC. As control plates, we prepared plates with a standard concentration of ampicillin of 100 μg/mL and plates without any antibiotics.

**Figure 9:**
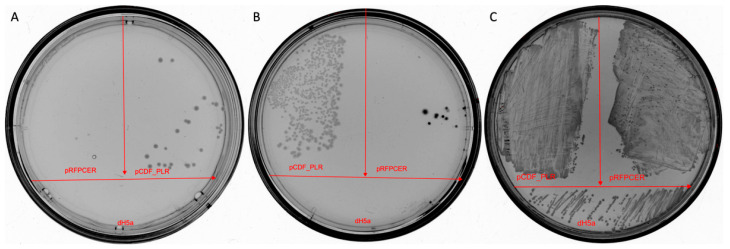
Verification of selective marker. On all plates, *E. coli* DH5α cells were plated after transformation of pCDF_PLR or pRFPCER, and as a control, non-transformed cells were used. (**A**) Plate with 1000 μg/mL of pentalenolactone, (**B**) plate with 100 μg/mL of ampicillin, (**C**) plate without antibiotics.

According to Figure 9A, only cells containing pCDF_PLR can grow on pentalenolactone. On ampicillin, cells with the original vector pRFPCER and cells with pCDF_PLR can grow (Figure 9B). On LB, all cells, including non-transformed variants, can grow (Figure 9C).

After initial confirmation that pentalenolactone can be a selectivity marker, we decided to test another hypothesis. Was it possible for cells to be protected from pentalenolactone by nonmutated *gapA* expression on a plasmid? To test this hypothesis, we made a construct that resembles pCDF_PLR but includes the wild-type *E. coli gapA* variant (without the substitution at position 175). We called this construction pCDF_GAPA. We transferred this construct to *E. coli* DH5α cells and measured the MIC in cells that contained the pCDF_PLR and pCDF_GAPA constructs (Table 3). The data obtained indicate that the pCDF_PLR construct containing the mutant version of the *gapA* gene provides resistance to pentalenolactone in a clear and unambiguous manner.

## 3. Discussion

Compounds of the pentalenolactone group exhibit antagonistic properties and are active against both Gram-positive and Gram-negative bacteria, as well as fungi and even some protozoa [5]. The mechanism of action of pentalenolactone has been identified as the inhibition of the glycolytic enzyme glyceraldehyde-3-phosphate dehydrogenase (GAPDH). The pentalenolactone compounds are competitive, covalent inhibitors of GAPDH, specifically alkylating Cys149 via an attack on the C9-C10 epoxide. Pentalenolactone initially binds to the enzyme in a reversible reaction that is competitively inhibited by the substrate glyceraldehyde-3-phosphate [18]. Then, it covalently and irreversibly binds to the thiol group of a cysteine residue in the active center of each of the four identical subunits [19].

*Streptomyces* sp. AP22 produces the antibiotic pentalenolactone, a highly specific inhibitor of glyceraldehyde-3-phosphate dehydrogenase (GAPDH). *Streptomyces* sp. AP22 contains a 12.5 kbp-long gene cluster containing 10 unidirectionally transcribed open reading frames corresponding to the putative biosynthetic operon of the sesquiterpene antibiotic pentalenolactone (Figure 6). This cluster contains nine genes responsible for pentalenolactone biosynthesis, and one gene gap1 provides self-resistance for the strain. The antibiotic synthesis cluster in *Streptomyces* AP22 is highly similar to *Streptomyces* NBC_01231 and *Streptomyces avermitilis* [20], which are well-known pentalenolactone producers.

As a rule, self-resistance in the pentalenolactone producer strain is due to the presence of two GAPDH isoenzymes: an inducible, pentalenolactone-insensitive form and a constitutive, pentalenolactone-sensitive form [21]. The molecular difference between pentalenolactone-insensitive GAPDH and all other GAPDH that protect pentalenolactone-insensitive GAPDH from the antibiotic was determined by Fröhlich et al. [22]. The pentalenolactone-insensitive GAPDH is expressed exclusively during pentalenolactone production [22], so it is highly regulated. The hairpin structures are probably elements that keep the gene in an inactive state during normal cell proliferation. The pentalenolactone-insensitive GAPDH differs from all closely related GAPDH by only a few amino acid residues, none of which are directly involved in catalysis or substrate binding. The overall amino acid composition is more similar to the GAPDH of thermophilic species than mesophilic species.

A comparison of GAPDH sequences in different species has shown that during evolution, a significant proportion of amino acid residues have changed little or not at all [23]. GAPDH is one of the most conserved proteins in bacteria and eukaryotes [24].

Antibiotic resistance can be caused by spontaneous mutations in bacterial DNA. The target site of antibiotics, such as ribosomal RNA or penicillin-binding proteins, can be affected by mutations in the target site. Mutations that confer resistance to *Staphylococcus aureus* against methicillin [25] are a classic example. In our case, this mutation occurred in the *gapA* gene (Glyceraldehyde-3-phosphate dehydrogenase A), as described in the literature for pentalenolactone [26].

A comparison of two bacterial GAPDH protein structures (wild type and mutated) that were generated using an AlphaFold program [27] has not revealed significant conformational changes that can be responsible for the appearance of tolerance of the mutated GAPDH to pentalenolactone, as it firstly suggested. Resistance is observed after substituting threonine with serine at position 175. Such mutations are not present in *Streptomyces* species that produce pentalenolactone, which has a natural resistance to antibiotics due to modified GAPDH enzymes. The only difference between wild-type and mutated *E. coli* GAPDH proteins is the lack of a methyl group, and it is unclear how resistance can arise. The main possible explanation is a slight change in hydrophobicity, which may possibly change the stability of the complex of pentalenolactone and the active site of GAPDH. This is also confirmed by the proximity of the mutated residue to the 150th cysteine (about 8 Å), which is located in the active center of the enzyme and targets the pentalenolactone.

In bacteria, ampicillin and kanamycin are the most frequently employed selection agents for maintaining plasmids with their respective resistance genes [28], beta-lactamase [29] and neomycin phosphotransferase II [30], respectively. In certain instances, it is not feasible to utilize this marker due to natural or accumulated resistance to this antibiotic in recipients [31]. The strain’s resistance to multiple antibiotics used in medicine may make transformation impossible in certain cases. In such cases, the use of antibiotics that are not used in clinical practice could be a workaround, but this does not always work [32]. Developing new markers of resilience is a good way to prevent this, but they are not frequently developed [28,33]. The problems mentioned earlier can be overcome with the help of the selection marker we proposed. The mutation we discovered offers us a 64-fold increase in resistance. Furthermore, this marker has a distinct quality—GAPDH [24] is a gene that is highly conserved in various organisms, making it a universal marker for a wide range of organisms.

## 4. Materials and Methods

### 4.1. Isolation of Strains

The strain was isolated from the soil of the Akhshtyr Gorge in Krasnodar Krai 43.517689° (N), 39.995838° (E). The isolation of candidate strains was performed by microbiological sowing on dense media “mineral agar №1 Gauze” of the following composition (g/L): starch—20, NaCl—0.5, K_2_HPO_4_—0.5, MgSO_4_—0.5, FeSO_4_—0.01, agar—20, and tap water; pH 7.2–7.4. Nystatin (250 µg/mL, Sigma-Aldrich, Rockville, MD, USA) and nalidixic acid (10 µg/mL, Sigma-Aldrich, USA) were added to all media to prevent the growth of fungi and Gram-negative bacteria, respectively. Rubomycin (10 μg/mL and 20 μg/mL, Sigma-Aldrich, USA) and streptomycin (2.5 μg/mL and 25 μg/mL, Sigma-Aldrich, USA) were also added to isolate members of “rare” genera of actinobacteria. Soil suspension was prepared and a series of 10-fold dilutions were made. The inoculated dense media were incubated at 28 °C for 7 days.

### 4.2. Screening of the Antimicrobial Potential

Screening for antibiotic activity in vivo was carried out using a fluorescence reporter system based on the *Escherichia coli* strain JW5503 ΔtolC with introduced plasmid pDualrep2, which allows us to determine the mechanism of action of the antibiotic simultaneously with the inhibition efficiency test of the antibiotic. In the presence of inhibitors of protein synthesis or molecules that disrupt DNA replication, the reporter strain after incubation (37 °C, 18 h) is able to produce fluorescent proteins RFP and Katushka2S, which are visualized in the ChemiDoc scanner (Bio-Rad, Hercules, CA, USA) in the Cy-3 and Cy-5 channels, respectively.

### 4.3. DNA Quality Control After Purification

DNA quality was evaluated by electrophoresis on 1% agarose gel and additionally spectrophotometrically using the Nanodrop 2000 spectrophotometer (Thermo Scientific, Waltham, MA, USA). According to the manufacturer’s recommendation, the A260/A280 acceptable ratio is 1.8–2.0, and the A260/A230 acceptable ratio is 2.0–2.2 for a DNA sample of good quality for the nanopore analysis. If the samples did not meet the given ratios, additional purification with the MagAttract HMW DNA Handbook (Qiagen, Germantown, MD, USA) kit was performed. The DNA concentration was then further scored by using the Qubit 3.0 fluorometer (Invitrogen, Thermo Fisher Scientific). Sample preparation for fluorometry was performed by using the Qudye dsDNA HS Assay kit (Lumiprobe, Moscow, Russia).

Fragment length detection was performed on 1% agarose gel with 70 µg/mL of ethidium bromide by using a ChemiDoc scanner (Bio-Rad, USA). Data analysis with the determination of the average fragment length was performed by using ImageLab v6.1 2020 by Bio-Rad Laboratories software.

### 4.4. Nanopore Sequencing

Nanopore sequencing was performed by Oxford Nanopore sequencing technology for genomic DNA by ligation by using the SQK-LSK109 protocol (Oxford Nanopore, Oxford, UK). The procedure of sample preparation was performed according to ref [34]. Oxford Nanopore’s instructions were followed for loading flow cells and DNA library preparation. The DNA library was then loaded on an R9.4.1 MinION Mk flow cell (Oxford Nanopore, UK) in accordance with the manufacturer’s protocol.

For the data collection for several strains in a single run, the additional barcoding step was performed before the adapter ligation procedure by using a Native Barcoding Expansion 1–12 (EXP-NBD104) kit (Oxford Nanopore, UK). The barcoding step was performed according to the manufacturer’s protocol, which is similar to the adapter ligation step and includes the barcode ligation and DNA purification steps. After barcoding ligation, an equimolar mixture of DNA samples was used in the adapter ligation step.

### 4.5. Data Analysis

We utilized a Guppy basecaller [35] to convert raw data in fast5 format to the basecalled data in fastq format. Flye 2.9 [10] was utilized for de novo genome assembly. All reads with the quality Q < 7.5 were excluded from the subsequent data analysis. The lengths feature of the sample were N50/N90 = 3431/1048. A total of 80 contigs were assembled in total with the largest one being 2.1 Mb. The mean coverage was 16. Contigs obtained de novo were polished with Medaka [Medaka: Sequence Correction Provided by ONT Research. Available online: https://github.com/nanoporetech/medaka (accessed on 1 November 2024)].

The phylogeny of the Streptomyces sp. AP22 strain was identified using NCBI Blastn (https://blast.ncbi.nlm.nih.gov/Blast.cgi#alnShow_1 (accessed on 1 November 2024)) and studied using the MLST approach based on an analysis of the gene encoding 16S and five housekeeping genes. Pentalenolactone biosynthetic gene clusters in the complete genome of strain Streptomyces sp. NBC_01231 (CP108521.1) were identified with the bacterial version of antiSMASH version 7.1.0 (https://antismash.secondarymetabolites.org/#!/start (accessed on 1 November 2024)). Homologous regions on each genome were identified using alignment tools of the SnapGene program.

The structures of wild-type and mutated GapA protein were gained using the AlphaFold program. An analysis of the protein structures was performed using PyMOL.

### 4.6. Phenotypic Characterization

Cultural characteristics of strain AP22 were observed in ISP 2–ISP 6 media as previously described [36] after cultivation for up to 14 days at 28 °C. The RAL Classic Standard was used to determine the designations of colony colors. The shape of spore chains and the spore surface of strain AP22 on ISP 3 after cultivation at 28 °C for 14 days were studied using scanning electron microscopy (JSM-6380LA, JEOL, Tokyo, Japan).

### 4.7. Purification and Identification of Active Compound in Cultural Broth of AP22

To obtain a sufficient amount of active compound, *Streptomyces* sp. AP22 was cultured in ten 750 mL Erlenmeyer flasks with 250 mL of Gauze-1 liquid nutrient medium (g/L: starch—20, NaCl—0.5, K_2_HPO_4_—0.5, MgSO_4_—0.5, FeSO_4_—0.01, and tap water; pH 7.2–7.4) at 28 °C for 7 days on a rocker at a rotation speed of 200 rpm. The dia-m company provided all the salts and starch.

Preparative purification of the antibiotic contained in the culture fluid was carried out by solid-phase extraction on an LPS-H-500 (Techsorbent, Perm, Russia) hydrophobic sorbent with a pore size of 120 μm, eluting with ascending concentrations of acetonitrile. The most active fraction was eluted with 10% acetonitrile and induced expression of the Katushka2S reporter protein. This fraction was evaporated in a vacuum concentrator (Labconco, Kansas City, MO, USA) at 37 °C to half of the original volume. The final purification of the active ingredient was performed by analytical HPLC under neutral and acidic conditions using an Agilent 1200 chromatograph (USA) on a Luna 5um C18(2) 100A column (Phenomenex, Torrance, CA, USA). The obtained fractions were again screened using the pDualrep2 system, and the fraction with the peak exhibiting antagonistic activity was selected. Then, the fraction was lyophilized and diluted with a 10% acetonitrile solution to a concentration of 20 mg/mL. 

The fraction with antibacterial activity corresponding to the individual peak on the chromatogram was collected to identify the active compound using ultra-performance liquid chromatography–electrospray ionization–high-resolution mass spectrometry (UPLC-ESI-HRMS). The analysis was performed on an UltiMate 3000 Acclaim RSLC HPLC system with a 120 C18 2.2 um 2.1 × 100 mm column, gradient of acetonitrile (0.1% FA) in water (0.1% FA) 5–95% for 10 min, connected to a Bruker maXis II 4G ETD (Thermo Scientific, USA). Spectra recording mode: ESI ionization mode, full scan from 100 to 1500 *m*/*z*, MS/MS with a selection of the three most intense ions, dissociation type: CID 10–40 eV, collision gas nitrogen.

### 4.8. Determination of Minimal Inhibitory Concentration (MIC)

For this assay, we used several strains: *E. coli* DH5α and *E. coli* ΔtolC SQ110, from our laboratory collection. Overnight cultures of this strain were diluted 1:1000 in an LB medium. A sterile 96-well plate was then loaded with 200 mL of the diluted inoculated cultural media, with an initial volume of 400 mL prior to serial dilution. A stock solution of an HPLC-purified AP22 sample (20 mg/mL) was added to initial wells, along with erythromycin (Ery, 5 mg/mL, Sigma-Aldrich, Rockville, MD, USA), which was used as a control for the experiment. In other wells, we did not add AP22 or Ery but added diluted inoculated LB-culture media, while the rest were left with LB media only as additional controls of sterility. A two-fold serial dilution was then carried out, with gentle mixing in each row. The plates were then incubated overnight at 37 °C with shaking at 200 rpm. Cell growth was measured at 590 nm using a microplate reader (VICTOR X5Light Plate Reader, PerkinElmer, Shelton, CT, USA). Three replicates were executed for all experiments, and the results are presented as those that were discovered in at least two replications. No discrepancies were greater than 2 times.

### 4.9. Selection of Resistant Clones

This experiment was performed to detect mutations in the *gapA* gene of the *E. coli* ΔtolC SQ110 strain that may confer resistance to the active compound of AP22. For this purpose, an *E. coli* ΔtolC SQ110 strain with only one of the seven ribosomal operons was used. Plates of LB agar were made with the addition of 3, 5, and 8 times higher concentrations than those shown to be minimally inhibitory concentrations on this strain. In total, 50 µL of the overnight *E. coli* ΔtolC SQ110 culture was then applied to a plate, and the inoculum was spread over the plate with a spatula and incubated for 10–12 h at 37 °C. After this time, the dishes were checked for colony growth and then genomic DNA was isolated from overnight cultures of resistant clones using a commercially available product Qiagen (USA) and sequenced using the primers gapA_F (5′-gagatataccATGACTATCAAAGTAGGTATCAAC-3′) and gapA_R (5′-ttttgcggccTTATTTGGAGATGTGAGC-3′). Lumiprobe (Russia) supplied primers.

### 4.10. Plasmids and Cloning

To create the construct pCDF_PLR, the vector backbone was amplified by high-fidelity PCR from the pRFPCER plasmid [9] using primers 5′-ctccaaataaGGCCGCAAAAATTAAAAATG-3′ and 5′-tgatagtcatGGTATATCTCCTTCTTTGAATC-3′. The *gapA^mut^ (T175S)* gene was amplified by PCR from the genome of the resistant *E. coli* strain with 5′-gagatataccATGACTATCAAAGTAGGTATCAAC-3′ forward and 5′-ttttgcggccTTATTTGGAGATGTGAGC-3′ reverse primers. The joining of two DNA fragments was performed with the NEBuilder^®^ HiFi DNA Assembly technique (NEB, USA). Lumiprobe (Russia) supplied primers.

The *E. coli* DH5α strain was used for DNA cloning. Sequences of intermediate products and final constructs were confirmed by sequencing with appropriate primers. Plasmid maps were visualized using the program Geneious Prime (version 2023.2.1).

## 5. Conclusions

In recent decades, genetic engineering has become an indispensable tool in modern biotechnology, contributing to the creation of new biological products and the improvement of existing ones. The research and development of genetically modified organisms with superior or novel functional properties is a fascinating trend in this field.

In this context, the antibiotic pentalenolactone is particularly interesting as a potential promising marker for genetically engineered constructs. The pentalenolactone resistance gene’s incorporation into multidrug-resistant pathogenic strains as a selective marker is highly interesting. Efficient selection and manipulation of these strains during the process will provide control over experiments and research aimed at developing new treatments for infections.

The findings are expected to provide new tools to create more effective treatments for infections, increasing resources in the fight against pathogens resistant to existing antibiotics. This development has the potential to be applied in various fields of medicine and biotechnology, contributing to the development of innovative methods of disease control and treatment.

## Figures and Tables

**Table 1:** Morphological properties of *Streptomyces* sp. AP22 and *Streptomyces scabiei* strains on different media. Data for *Streptomyces scabiei* (NCPPB 4066, DSM 41658) are from DSMZ catalogue (https://www.dsmz.de/collection/catalogue/microorganisms/catalogue (accessed on 1 November 2024)).

Culture Attributes	*Streptomyces* sp. AP22					*Streptomyces scabiei*				
	ISP2	ISP3	ISP4	ISP5	ISP6	ISP2	ISP3	ISP4	ISP5	ISP6
Growth	++	++	+++	+++	+	++	++	++	++	-
Coloration of aerial mycelium	Light gray	Light gray	White	Grey	Colorless	Grey	Grey	White	Grey	Colorless
Substrate mycelium coloration Substrate mycelium coloration	Brown	Light yellow	White	Light yellow	Colorless	Black	Colorless	Colorless	Fir green	Colorless

**Table 2:** Minimal inhibitory concentration of pentalenolactone for resistant clone.

Antibiotics	MIC for *E. coli* SQ110DTC, μg/mL	MIC for *E. coli* SQ110DTC Resistant Clone, μg/mL
Pentalenolactone	25	200
Erythromycin	3.13	3.13

**Table 3:** Difference in MIC between *E. coli* DH5α transformed with pCDF_PLR and pCDF_GAPA constructs.

Antibiotics	MIC on *E. coli* DH5α Cells with pCDF_PLR Vector (Mutant gapA), μg/mL	MIC on *E. coli* DH5α Cells with pCDF_GAPA Vector (WT gapA), μg/mL
Pentalenolactone	3200	50
Erythromycin	125	125

## Data Availability

The annotated pentalenolactone synthesis cluster sequence of *Streptomycetes* sp. AP22 described in this paper was deposited to GenBank and is available under the following accessions: Gap1 PQ529460, pnt1 PQ529461, pnt2 PQ529462, pnt3 PQ529463, pnt4 PQ529464, pnt5 PQ529465, pnt6 PQ529466, pnt7 PQ529467, pnt8 PQ529468, and pnt9 PQ529469. Bacterial housekeeping genes, used to build a phylogenetic tree, are available under the following accessions: 16S rRNA PQ498873, atpD PQ522348, gyrB PQ522349, recA PQ52235, rpoB PQ522350, and trpB PQ522351.
